# Antibiotic consumption in Belgian acute care hospitals: analysis of the surveillance methodology, consumption evolution 2003 to 2016 and future perspectives

**DOI:** 10.2807/1560-7917.ES.2019.24.46.1900098

**Published:** 2019-11-14

**Authors:** Eline Vandael, Koen Magerman, Samuel Coenen, Herman Goossens, Boudewijn Catry

**Affiliations:** 1 Healthcare-associated infections and antimicrobial resistance, Sciensano, Brussels, Belgium; 2 Belgian Antibiotic Policy Coordination Committee (BAPCOC), Brussels, Belgium; 3 Jessa Hospital, Hasselt, Belgium; 4 Department of Microbiology, UHasselt, Hasselt, Belgium; 5 Laboratory of Medical Microbiology, Vaccine & Infectious Disease Institute (VAXINFECTIO), University of Antwerp, Antwerp, Belgium; 6 Faculty of Medicine, Université libre de Bruxelles (ULB), Brussels, Belgium

**Keywords:** antibiotic consumption, antimicrobial use, surveillance, hospitals, Belgium

## Abstract

**Background:**

Studies have demonstrated the link between antimicrobial consumption and the development of antimicrobial resistance. Surveillance of antimicrobial consumption is an action point of the European Commission’s ‘One Health Action Plan Against Antimicrobial Resistance’.

**Aim:**

This study aims to compare two methodologies for antibiotic consumption surveillance, investigate the 14-year evolution of antibiotic consumption in Belgian acute care hospitals and discuss future perspectives.

**Methods:**

We compared self-reported data (old methodology) and reimbursement data (new methodology) of national antibiotic consumption surveillance in hospitals. Descriptive analyses were performed on the reimbursement data collected per year and per trimester (2003–2016), per hospital and per unit. Antibiotic consumption was compared with European Surveillance of Antimicrobial Consumption Network (ESAC-Net) results.

**Results:**

The median differences for defined daily doses (DDDs)/1,000 patient days and DDDs/1,000 admissions were 3.09% and 3.94% when comparing the old vs new methodology. Based on reimbursement data, the median antibiotic consumption in 2016 in 102 Belgian acute care hospitals was 577.1 DDDs/1,000 patient days and 3,890.3 DDDs/1,000 admissions with high variation between hospitals (interquartile ranges (IQR): 511.3–655.0 and 3,450.0–4,400.5, respectively), and similar to 2015. Based on DDDs/1,000 patient days, the magnitude of consumption is comparable with the Netherlands, Denmark and Sweden, but is higher when based on DDDs/1,000 admissions.

**Conclusion:**

Antibiotic consumption in Belgian acute care hospitals has remained overall stable over time. However, the high variation across hospitals should be further investigated. This surveillance data could be used for benchmarking and assessing interventions to improve antibiotic consumption in these hospitals.

## Introduction

Antimicrobial resistance, which is defined as ‘the ability of a microorganism to resist the action of one or more antimicrobial agents’ [1], is a serious worldwide threat for public health. In Europe, ca 25,000 deaths a year are caused by infections with bacteria that are resistant to antimicrobials. Moreover, antimicrobial resistance leads to a high morbidity and additional costs for the society. According to a report published in 2009, these costs were estimated to be at least EUR 1.5 billion per year in the European Union (EU) [2]. Joint international efforts are needed to control this threat and to prevent infections from becoming a top cause of mortality again [3,4].

Several studies have demonstrated the link between antimicrobial consumption and the emergence of infections with antimicrobial resistant invasive pathogens [5-8], which underlines the importance of responsible and prudent antimicrobial use. Surveillance of antimicrobial consumption is one of the action points of the European Commission’s ‘One Health Action Plan against Antimicrobial Resistance’ [3].

Belgium has played a leading role in the development and implementation of surveillance systems on antimicrobial consumption and resistance, which included coordinating the European Surveillance of Antimicrobial Consumption, now the European Surveillance of Antimicrobial Consumption Network (ESAC-Net) organised by the European Centre for Disease Prevention and Control (ECDC), from 2001 to 2011. Domestically, in 1999, the Belgian Antibiotic Policy Coordination Committee (BAPCOC), an advisory and executing body at federal level, was launched to mitigate the spread of antimicrobial resistance and optimise the antimicrobial use in different settings, primary care, hospitals, long-term care facilities and animal husbandry [9]. Since 2007, a national surveillance system for the consumption of systemic antibacterial agents, under the management of the Scientific Institute of Public Health (WIV-ISP), now Sciensano, has been implemented in acute care and large chronic care hospitals, i.e. chronic care hospitals with 150 or more patient beds. Until 2014, hospitals were obligated to annually upload their consumption data on a web-based data collection application called NSIHweb (www.nsih.be). Since 2014, a new methodology has been developed using reimbursement data of the national health insurance institute to align data collection activities with the Belgian law of the ‘only once’ principle of data collection (Royal Decree 5 May 2014) and with the goal to harmonise data collection in different hospitals. This optimised surveillance system is called Belgian Hospitals - Surveillance of Antimicrobial Consumption (BeH-SAC).

The objective of this study is to compare the old and new methodology for the national surveillance of antibiotic consumption, and to describe the 14-year evolution (2003–2016) of antibiotic use in Belgian acute care hospitals using the new national surveillance system, BeH-SAC.

## Methods

### Data collection

#### New surveillance system based on reimbursement data

Reimbursement data on antibiotic consumption were collected as part of BeH-SAC from the Research, Development and Quality department of the National Institute for Health and Disability Insurance (NIHDI) in Belgium. Consequently, any non-reimbursed, off-label use of antibiotics or import of antibiotics from other countries could not be taken into account. In 2016, 98.6% of the Belgian population had health insurance and are therefore included in the reimbursement data [10].

Both numerator (number of consumed units per drug) and denominator (admissions and number of patient days, with day of admission and discharge included) data were requested for the period 2003 to 2016 for Belgian acute care, chronic care and psychiatric hospitals. In 2016, 102 acute care hospitals in Belgium were included in the reimbursement data (54 in Flanders; 36 in Wallonia; 12 in Brussels). An overview table with the number and characteristics of the included acute care hospitals for all years can be found in the supplementary data (Supplementary Table S1). The number of admissions was only available for the period 2008 to 2016. The data were delivered per year, per trimester, per hospital (based on a unique NIHDI number) and per unit (including surgery, internal medicine, geriatrics, paediatrics, intensive and non-intensive neonatology, maternity, infectious disease, burn unit, intensive care (ICU), specialised care, psychiatry, and surgical one-day hospitalisations). For our study, psychiatry wards and one-day hospitalisation admissions were excluded to obtain full comparability with the old methodology [11].

To classify the antimicrobials drugs, the Anatomical Therapeutic Chemical (ATC) classification of the World Health Organization (WHO) Collaborating Centre for Drugs Statistics Methodology was used [12]. The following ATC codes are currently included in BeH-SAC surveillance: A07A (Intestinal anti-infectives), D01BA (Antifungals for systemic use), J01 (Antibacterials for systemic use), J02 (Antimycotics for systemic use), P01AB (Nitroimidazole derivatives), J04A (Drugs for treatment of tuberculosis) and J05 (Antivirals for systemic use).

#### Old surveillance system based on self-reported data

The old methodology was based on self-reported data from hospitals (both numerator and denominator data), collected via the web-based data collection application NSIHweb [13]. Table 1 presents an overview of the major differences between the old and the new antimicrobial surveillance methodologies.

**Table 1:** Comparison of old and new methodologies for national surveillance of antimicrobial consumption in hospitals, Belgium

Component	Old methodology (NSIHweb) [13]	New methodology (BeH-SAC) [11]
Data source	Self-reported sales data (numerator and denominator) by hospitals, mandatory surveillance Participation rate: ca 88% in 2012 [13]	Reimbursement data, which covers ca 98.6% of the Belgian population with health insurance in 2016 [10]
Indicators	DDDs/1,000 patient days, DDDs/1,000 admissions	DDDs/1,000 patient days, DDDs/1,000 admissions
Period	2007–14 (mandatory until 2013) Per year and per trimester (voluntary)	DDDs/1,000 patient days: 2003–most recent year DDDs/1,000 admissions: 2008–most recent year Per year and per trimester
Drugs (ATC codes)^a^	A07A, D01BA, J01, J02, P01AB, J04A	A07A, D01BA, J01, J02, P01AB, J04A, J05
Hospitals^a^	Acute care hospitals and chronic care hospitals (≥ 150 beds) Per merger^b^ and per site (voluntary)	Acute care, chronic care and psychiatric hospitals Per merger^b^
Hospital units	Total hospital use: psychiatric beds only included in the numerator (excluded in the denominator) and one day hospitalisations excluded. Specific results for non-paediatric wards, paediatric wards, ICU (voluntary) and hematology-oncology (voluntary).	Total hospital use: data on psychiatry and surgical one day hospitalisations available, but excluded in the current study to ensure comparability with the old methodology^c^. Specific results for surgery, internal medicine, geriatrics, paediatrics, intensive and non-intensive neonatology, maternity, infectious disease, burn unit, ICU, specialised care, psychiatry and surgical one day hospitalisations.
Feedback reporting	Interactive feedback reports per hospital with benchmarking Platform: NSIHweb	Interactive feedback reports per hospital with benchmarking Platform: Healthstat

ATC: anatomical therapeutic chemical classification; BeH-SAC: Belgian Hospitals – Surveillance of Antimicrobial Consumption; DDD: defined daily dose; ICU: intensive care unit.

^a^ In the current study, only antibiotic consumption (ATC code J01) and acute care hospitals were included.

^b^ Hospital sites that are grouped together.

^c^ Full results (including on psychiatry wards and surgical one-day hospitalisation) are available in the interactive reports on www.healthstat.be.

### Data analysis

In this study, we focus on the consumption of antibiotics, i.e. antibacterials for systemic use (ATC code J01) in acute care hospitals. In contrast to ESAC-Net, where consumption in hospitals is expressed on a national level in defined daily doses (DDDs) per 1,000 inhabitants/day (DIDs), also the following indicators are used: DDDs/1,000 admissions and DDDs/1,000 patient days [14,15]. The number of packages per drug were converted into DDDs in line with the DDD/ATC classification of the WHO Collaborating Centre for Drugs Statistics Methodology, version February 2018 [12]. Numerator and denominator data could be linked based on each hospital’s unique NIHDI number.

Hospitals were classified per type (primary, secondary, tertiary and specialised) in accordance with the European Centre for Disease Prevention and Control (ECDC) recommendations [16] and based on the list of hospitals provided by the Federal Public Service Health, Food Chain Safety and Environment (Dienst Datamanagement - Directoraat-Generaal Gezondheidszorg, version 2/2018).

### Comparison of two surveillance systems

The median difference and interquartile range (IQR) in the results on overall antibiotic consumption (J01) per hospital between the new and the old methodology was calculated for years with overlapping data (2007–2014) for all acute care hospitals with data available. The number of hospitals covered per year was as follows: 52 in 2007 (pilot year); 93 in 2008; 94 in 2009; 95 in 2010; 94 in 2011; 90 in 2012; 81 in 2013; and 22 in 2014 (transition year).

A SWOT-analysis (strengths, weaknesses, opportunities and threats) of the new methodology was performed.

Trends over time were calculated in both databases with the Mann-Kendall test. P values ≤ 0.05 were considered statistically significant.

### Analyses based on data from the new surveillance system

To study antibiotic consumption in Belgian acute care hospitals, both longitudinal and compositional analyses were done on data of BeH-SAC (version database August 2018). Median and interquartile range (IQR) were calculated where appropriate. Boxplots were used to present the evolution of the consumption and the variability between hospitals. Total antibiotic use in Belgian acute care hospitals was compared with ESAC-Net data and national report data from the Netherlands, Denmark, Sweden and France (north-western European countries for which these reports with recent data were easily available), using the indicators DIDs, DDDs/1,000 patient days (considered the same as DDDs/1,000 occupied bed-days) and DDDs/1,000 admissions. The consumption of the broad-spectrum antibiotic subclasses J01CR, J01DD/DE, J01DH, J01MA and J01XA were investigated more in detail. All data analyses were performed with SAS Enterprise Guide version 7.1.

### Ethical statement

No ethical approval was obtained for this study since no patient-level data were collected.

## Results

### Comparison of the old and new methodologies’ results

In the comparison of the antibiotic consumption rates per hospital and per year calculated with data from the old and new methodologies, a median difference of 3.09% in DDDs/1,000 patient days (IQR: 1.28–8.02) and a median difference of 3.94% in DDDs/1,000 admissions (IQR: 1.66–13.24) was found. For antibiotic consumption expressed as DDDs/1,000 patient days and DDDs/1,000 admissions a difference of more than 50% was detected for 1 or more years for 13 and 15 hospitals, respectively. Trend-analysis showed a significant increasing trend in antibiotic consumption in DDDs/1,000 patient days in both the old (p = 0.036, Kendall’s τ_b_: 0.060) and the new methodology (p = 0.001, Kendall’s τ_b_: 0.092). For antibiotic consumption in DDDs/1,000 admissions, no significant trend was found in either database (old methodology: p = 0.272; Kendall’s τ_b_: −0.033; new methodology: p = 0.281; Kendall’s τ_b_: −0.032).

Supplementary Table S2 presents the SWOT-analysis of BeH-SAC in which different aspects of this new methodology are summarised.

### Analysis of antibiotic consumption in acute care hospitals

**Figure 1:**
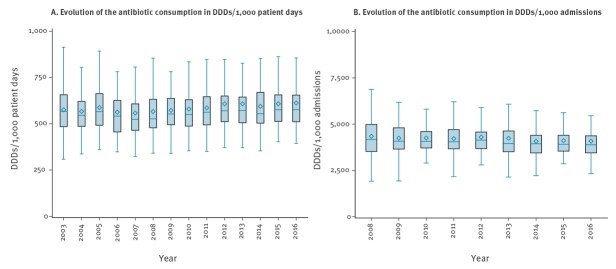
Evolution of the consumption of antibacterials for systemic use (ATC code J01) in acute care hospitals, Belgium, 2003–2016

ATC: Anatomical Therapeutic Chemical classification; DDD: defined daily doses; IQR: interquartile range.

The evolution of antibiotic consumption in acute care hospitals is presented in Figure 1. Of notice, the mean length of stay in these hospitals decreased from 7.72 days in 2008, which is when data became available, to 6.66 days in 2016.

Legend boxplot: the upper whisker indicates the maximum value (that is not an outlier, 1.5xIQR); the top of the box indicates the 75th percentile (P75); the middle line of the box indicates the median; the diamond indicates the mean; the bottom of the box indicates the 25th percentile (P25); and the bottom whisker indicates the minimum value (that is not an outlier, 1.5xIQR).

For results based on the number of admissions (B), we only have data starting from 2008.

The median antibiotic consumption in acute care hospitals in 2016 was 577.1 DDDs/1,000 patient days, which is an increase of 1.76% (10.0 DDDs/1,000 patient days) compared to 2003 (567.1 DDDs/1,000 patient days) and an increase of 0.14% (0.8 DDDs/1,000 patient days) compared to 2015 (576.3 DDDs/1,000 patient days). A significant increasing trend was found for antibiotic consumption in DDDs/1,000 patients days for the period 2003 to 2016 (p < 0.001; Kendall’s τ_b_: 0.068) and the period 2007 to 2016 (p < 0.001; Kendall’s τ_b_: 0.097).

Expressed in DDDs/1,000 admissions, the median antibiotic consumption in 2016 was 3,890.3. This is a decrease of 6.81% (−284.2 DDDs/1,000 admissions) in comparison with 2008 (4,174.5 DDDs/1,000 admissions) and a decrease of 0.57% (−22.2 DDDs/1,000 admissions) in comparison with 2015 (3,912.5 DDDs/1,000 admissions). A significant decreasing trend was detected for antibiotic consumption in DDDs/1,000 admissions for the period 2008 to 2016 (p = 0.002; Kendall’s τ_b_: −0.073).

**Figure 2:**
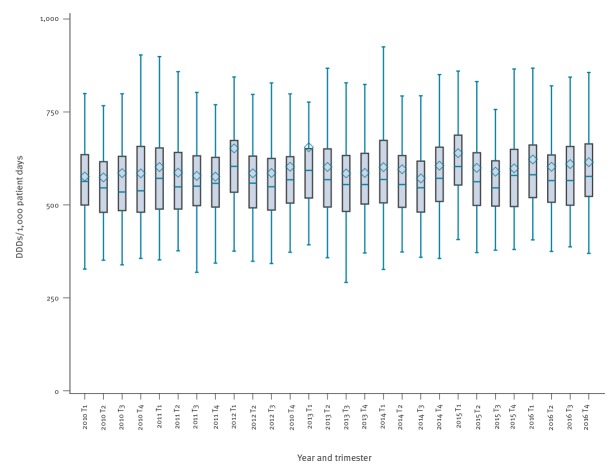
Evolution of the consumption of antibacterials for systemic use (ATC code J01) per trimester in acute care hospitals, Belgium, 2010–2016

ATC: Anatomical Therapeutic Chemical classification; DDD: defined daily doses; IQR: interquartile range.

T1: trimester 1, January-March; T2: trimester 2, April-June; T3: trimester 3, July-September; T4: trimester 4, October-December.

Little seasonal variation in antibiotic consumption (< 10% difference in median use between autumn-winter (trimester 1 and 4) and spring-summer (trimester 2 and 3)) was detected (Figure 2).

Legend boxplot: the upper whisker indicates the maximum value (that is not an outlier, 1.5xIQR); the top of the box indicates the 75th percentile (P75); the middle line of the box indicates the median; the diamond indicates the mean; the bottom of the box indicates the 25th percentile (P25); and the bottom whisker indicates the minimum value (that is not an outlier, 1.5xIQR).

The median antibiotic consumption in ICUs in 2016 was 1,261.0 DDDs/1,000 patient days, which is an increase of  5.25% (62.9 DDDs/1,000 patient days) in comparison with 2003 (1,198.1 DDDs/1,000 patient days). Median antibiotic consumption in ICUs in 2016 was twice as high as the overall median consumption (ICU included) in acute care hospitals (577.1 DDDs/1,000 patient days) (Supplementary Figure S1). More details about antibiotic consumption in other hospital units are available in Supplementary Table S3.

Antibiotic consumption in tertiary hospitals was substantially higher than in the other hospitals, with a median consumption of 715.0 DDDs/1,000 patient days (IQR: 702.1–858.3) in 2016 (Supplementary Figure S2). For the same year, the median antibiotic consumption in primary and secondary hospitals was 568.1 (IQR: 511.3–636.1) and 571.8 DDDs/1,000 patient days (IQR: 504.0–600.9), respectively. Between 2010 and 2016, increases in consumption in DDDs/1,000 patient days per hospital type were: 4.55% in tertiary hospitals; 8.21% in secondary hospitals and 6.29% in primary hospitals. Consumption evolution per hospital region and per hospital size is presented in Supplementary Figures S3 and S4.

Table 2 presents the median consumption per antibiotic subclass in 2016 (ATC level 4), the percentage of each subclass and the change in comparison with 2003 and 2015 in DDDs/1,000 patients days, or 2008 in DDDs/1,000 admissions. ‘Combinations of penicillins with beta-lactamase inhibitors’ (J01CR) was the most used subclass at 37.0% of the median consumption followed by ‘Fluoroquinolones’ (J01MA) at 11.1% (Table 2). In comparison with 2015, the median consumption in these subclasses decreased by 2.9% and 3.8%, respectively. The largest decreases in use from 2015 to 2016, were found for the subclasses ‘Monobactams’ (J01DF) (−24.5%) and ‘Amphenicols’ (J01BA) (−8.9%). On the other hand, the largest consumption increases were seen for ‘Polymyxins’ (J01XB) (40.0%), ‘Glycopeptide antibacterials’ (J01XA) (11.0%) and ‘Beta-lactamase-sensitive penicillins’ (J01CE) (8.9%). A stacked bar plot with the consumption of the most important antibiotic subclasses is presented in Supplementary Figure S5. In 2016 in 62.4% of the total DDDs for J01, the antibiotic agent was administered via a parental route (oral: 37.0%, inhalation: 0.6%, other: 0.1%). Amoxicillin in combination with a beta-lactamase inhibitor (J01CR02), ciprofloxacin (J01MA02) and cefazolin (J01DB04) were the most frequently used products.

**Table 2:** Overview of trends in consumption of antibacterials for systemic use (ATC J01) per drug class (ATC level 4) in acute care hospitals, Belgium, 2003–2016

ATC code (level 4)	Antibacterial subclass	Number of hospitals	DDDs/1,000 patient days				DDDs/1,000 admissions		
			Median consumption in 2016 over all hospitals (DDDs/1,000 patient days)	Percent of the median consumption in 2016 (%)	Change (%) 2003–16	Change (%) 2015–16	Median consumption in 2016 over all hospitals (DDDs/1,000 admissions)	Percent of the median J01 consumption in 2016 (%)	Change (%) 2008–16
J01AA	Tetracyclines	102	2.44	0.42	−11.98	−1.98	15.78	0.41	−19.78
J01BA	Amphenicols	90	2.18	0.38	−42.23	−8.93	14.92	0.38	−8.24
J01CA	Penicillins with extended spectrum	102	40.07	6.94	86.44	4.07	267.10	6.87	26.12
J01CE	Beta-lactamase sensitive penicillins	99	5.89	1.02	30.43	8.88	37.66	0.97	8.53
J01CF	Beta-lactamase resistant penicillins	102	24.21	4.20	47.04	5.68	158.32	4.07	12.14
J01CR	Combinations of penicillins, including beta-lactamase inhibitors	102	213.77	37.04	16.91	−2.91	1447.67	37.21	−11.58
J01DB	First-generation cephalosporins	102	38.84	6.73	30.96	−0.55	263.84	6.78	1.05
J01DC	Second-generation cephalosporins	102	15.06	2.61	−56.32	−4.34	95.07	2.44	−30.22
J01DD	Third-generation cephalosporins	102	26.01	4.51	6.74	0.46	169.15	4.35	1.26
J01DE	Fourth-generation cephalosporins	80	3.13	0.54	−83.83	0.57	19.15	0.49	−47.45
J01DF	Monobactams	73	0.40	0.07	−76.74	−24.53	3.10	0.08	−31.42
J01DH	Carbapenems	102	19.16	3.32	60.09	5.88	136.54	3.51	19.93
J01EE	Combinations of sulfonamides and trimethoprim, including derivatives	102	6.73	1.17	17.09	1.64	46.45	1.19	−5.38
J01FA	Macrolides	102	18.85	3.27	6.30	2.91	129.60	3.33	20.66
J01FF	Lincosamides	102	10.63	1.84	69.65	5.04	67.07	1.72	31.95
J01GB	Aminoglycosides	102	6.74	1.17	−64.12	−6.65	46.09	1.18	−50.17
J01MA	Fluoroquinolones	102	64.22	11.13	−15.66	−3.82	439.91	11.31	−16.05
J01XA	Glycopeptide antibacterials	102	9.25	1.60	24.43	10.97	59.00	1.52	1.34
J01XB	Polymyxins	78	1.19	0.21	340.74	40.00	7.76	0.20	12.63
J01XD	Imidazole derivatives	98	7.26	1.26	−9.34	−7.70	49.55	1.27	−6.86
J01XE	Nitrofuran derivatives	102	9.85	1.71	11.66	−2.48	68.34	1.76	−10.17
J01XX	Other antibacterials	102	3.08	0.53	107.88	−0.35	20.68	0.53	30.47

ATC: Anatomical Therapeutic Chemical classification; DDD: defined daily dose.

Antibacterial subclasses for which there was no consumption are not shown.

Based on the results expressed in DIDs, consumption in Belgian hospitals (1.95 DIDs) was higher than in the Netherlands (0.98 DIDs) and Sweden (1.65 DIDs), similar to Denmark (1.99 DIDs) and lower than France (2.19 DIDs) (Table 3). When expressed in DDDs/1,000 admissions, antibiotic consumption was remarkably higher in Belgium than the Netherlands, Denmark, and Sweden, yet lower when described as DDDs/1,000 patient days. Looking at specific broad-spectrum antibiotic subclasses, the consumption of ‘Combinations of penicillins including beta-lactamase inhibitors’ (J01CR) (34% of the total J01 consumption) was considerably higher in Belgium hospitals in comparison with the Netherlands, Denmark and Sweden, but the consumption of ‘Third/fourth-generation cephalosporins’ (J01DD/DE), ‘Carbapenems’ (J01DH), ‘Fluoroquinolones’ (J01MA) and ‘Glycopeptides’ (J01XA) was similar.

**Table 3:** Comparison of the total antibiotic consumption (ATC J01) and the consumption of antibiotic subclasses (J01CR, J01DD/DE, J01DH, J01MA, J01XA) in acute care hospitals in five European countries, 2015–2016

Year				Belgium^a^			Netherlands [27]^b^		Denmark [22]^c^		Sweden [28]^d^		France [23]^e^	
				2016			2015		2016		2016		2016	
**Antibiotics for systemic use (ATC J01)**														
DIDs ^f^				1.95^g^			0.98		1.99		1.65		2.19	
DDDs/1,000 pd				606			779		1,000		673		442	
DDDs/1,000 adm				4,088			3,301		3,105		2,972		NA	
**Combinations of penicillins, including beta-lactamase inhibitors (ATC J01CR)**														
DDDs/1,000 pd	% J01			208		34.3%	143	18.4%	180	18.0%	72	10.7%	NA	NA
DDDs/1,000 adm	% J01			1,403		34.3%	NA	NA	554	17.8%	318	10.7%	NA	NA
**Third/fourth-generation cephalosporins (ATC J01DD / J01DE)**														
DDDs/1,000 pd	% J01			30/7		5.0%/1.2%	55/0	7.1%/0%	10/0	1.0%/0%	NA	NA	NA	NA
DDDs/1,000 adm	% J01			199/58		4.9%/1.4%	NA	NA	32/0	1.0%/0%	NA	NA	NA	NA
**Carbapanems (ATC J01DH)**														
DDDs/1,000 pd		% J01	24		4.0%		17	2.2%	39	3.9%	31	4.6%	NA	NA
DDDs/1,000 adm		% J01	163		4.0%		NA	NA	119	3.8%	136	4.6%	NA	NA
**Fluoroquinolones (ATC J01MA)**														
DDDs/1,000 pd		% J01	66		10.9%		84	10.8%	81	8.1%	65	9.7%	NA	NA
DDDs/1,000 adm		% J01	448		11.0%		NA	NA	249	8.0%	287	9.7%	NA	NA
**Glycopeptides (ATC J01XA)**														
DDDs/1,000 pd		% J01	13		2.1%		16	2.1%	11	1.1%	11	1.6%	NA	NA
DDDs/1,000 adm		% J01	89		2.2%		NA	NA	32	1.0%	46	1.5%	NA	NA

ATC: Anatomical Therapeutic Chemical adm: admissions; DDD: defined daily dose; DIDs: DDDs/1,000 inhabitants/day; NA: not available; pd: patient days.

^a^ Belgium data sources: reimbursement data from acute care hospitals. Psychiatric wards and one-day hospitalisations excluded.

^b^ Netherlands: self-reported sales data by hospital pharmacies from acute care hospitals. All inpatient wards and day care centres included.

^c^ Denmark: sales data from public somatic hospitals. Private hospitals and psychiatric wards excluded. Results reported in DDDs/1,000 occupied bed days considered equivalent to DDDs/1,000 patient days.

^d^ Sweden: sales data from acute care hospitals. No exclusion of wards reported. Denominators (patient days and admissions) from 2015 were used.

^e^ France: sales data from all hospitals in France. No exclusion of wards reported.

^f^ Data from European Surveillance of Antimicrobial Consumption Network (ESAC-Net) [24]; consumption for the total hospital sector, extrapolated to 100% for reimbursement data.

^g^ Corrections performed based on a validation of the Belgian hospital data in ESAC-Net, leading to a different result than reported in the ESAC-Net 2016 report.

## Discussion

This study describes antibiotic consumption in Belgian acute care hospital using a recently optimised surveillance method. Major strengths are an extended observation period (2003–2016) and coverage (ca 99% of the Belgian population) as well as the availability of a detailed variable list for consumption data (e.g. per hospital unit, per trimester). As such, an in depth investigation over time is possible. Different indicators were used to express this consumption and they were compared with the results of the old surveillance system and results reported by other European countries.

Main advantages of the new methodology are the decreased registration load for the hospitals and the increased uniformity of the data collection across different hospitals. Contrary to the previous methodology, which was based on self-reported consumption data by hospitals, the current system uses NIHDI reimbursement data. A comparison between data obtained by the old and new methodology for years with overlapping data, indicated a small overall difference in the antibiotic consumption (median difference < 5%). Nevertheless, there were some large differences (> 50%) underlining the heterogeneity of the old methodology and the improved uniformity using BeH-SAC. In 2016, 98.6% of the Belgian population had a health insurance and hence are included in these reimbursement data [10]. Consequently, the underestimation by not taking into account non-reimbursed antibiotic use could be considered as negligible for trend evolutions.

The use of DDD as indicator can be seen as a limitation of both methods. Especially in paediatrics departments or in patients with special dose adjustments e.g. those with kidney failure, DDD is not an accurate indicator as it can lead to over- or underestimations of the dosing. Additionally, DDDs are not always in line with the actual doses used in Belgian hospitals, e.g. for amoxicillin the DDD should have been 1 g (the DDD version 2018 used in this study; in 2019, the DDD changed to 1.5 g), while the daily dose in practice is 3 to 4 g. Days of treatment (DOT) has been proposed as an alternative indicator, but this is currently not possible for most Belgian hospitals. DDD is still the most used and recognised indicator for antibiotic consumption worldwide. It allows, based on aggregated hospital data, to follow the evolution of consumption in a hospital and to benchmark with other institutions. It can be useful to also report the recommended daily dose in line with the national guidelines, alongside DDD (see future perspectives). Nationally recommended daily doses are not internationally comparable, but can help to interpret the consumption data on a national and local level [17-20].

A limitation with regards to the new method is that adaptations in reimbursement data (additional data or corrections) can still occur until 2 years after the first data delivery. Although big changes are not to be expected, the data of 2015–2016 therefore remain preliminary. A disadvantage of using reimbursement data in comparison with self-reported data is the larger delay (ca 12 months) in the data collection and consequently the feedback to the local hospitals. Further, based on the reimbursement data, we assume that the whole drug unit/ampoule is consumed, while this may not always be the case e.g. owed to individual dosing based on weight, which can lead to an overestimation of the consumption.

In France, Henard et al. compared three national surveillance systems for the antibiotic use in hospitals already in 2014, using both qualitative and quantitative analyses. They concluded that the three databases were heterogeneous in terms of objectives, data collection and results. Hence they recommended the development and implementation of one national instrument which would allow: (i) automated data collection to lower the work load, (ii) collection of data on different levels (i.e. national, regional, hospital and unit level), as well as (iii) benchmarking and rapid local feedback [21]. BeH-SAC adheres to the first three of these recommendations. Due to the current delay in reimbursement data, rapid local feedback is not possible.

Antibiotic consumption in Belgian acute care hospitals remained stable over time (minor increase in DDDs/1,000 patient days and minor decrease in DDDs/1,000 admissions, although significant) with a median consumption in 2016 of 577.1 DDDs/1,000 patient days and 3,890.3 DDDs /1,000 admissions. This means that in general, on half of the days, patients are receiving antibiotics. In reality, this will be lower as some patients received higher doses than the DDD, or received a combination of several antibiotics. The boxplots indicate that there is a high variation between hospitals and this variation also remained stable over time. The gap between high and low antibiotic-consuming hospitals remained evident when stratified per hospital type (tertiary, secondary, primary). The evolution towards shorter hospital stay in Belgian hospitals might explain the minor increase in DDDs/1,000 patient days over time, and the minor decrease in DDDs/1,000 admissions. This was also reported by other authors [14,15] and in other European countries [22,23].

In 2016, antibiotic consumption in Belgian hospitals was similar to 2015. The same trend was reported in ESAC-Net, for which the same numerators but different denominators were used (1.97 and 1.95 DID in 2015 and 2016, respectively) [24]. Antibiotic consumption was substantially higher in tertiary hospitals. Within the hospitals, the highest consumption was found in ICU departments. This can be explained by the more severe and complex diagnoses with a higher infection risk on ICUs. ‘Penicillins in combination with beta-lactamase inhibitors’ (J01CR) was the most prescribed antibiotic subclass in Belgian hospitals (37.0% of the median antibiotic consumption), followed by ‘Fluoroquinolones’ (J01MA, 11.1%). Since 2007, antibiotic management teams are implemented in all Belgian acute care hospitals [25]. No clear difference in antibiotic consumption was detected after this implementation. This lack of notable impact of these teams on antibiotic consumption was also previously reported by Lambert et al. [26]. Over the last 5 years (2013–2018), BAPCOC implemented interventions on the quality of antibiotic prescribing in hospitals focusing on specific topics, e.g. surgical prophylaxis. Yet, the impact of these interventions is difficult to assess based on the currently available consumption data.

As depicted in Table 3, total antibiotic consumption (J01) in Belgian acute care hospitals is comparable with other European countries with a restrictive antibiotic policy, i.e. the Netherlands, Denmark, Sweden and France [22,23,27,28]. Although differences in the methodology of national surveillance systems should be taken into account when interpreting these results. Three indicators were used to express the consumption: DIDs (used in ESAC-Net), DDDs/1,000 patient days (or occupied bed-days), and DDDs/1,000 admissions. Comparison may be difficult as the analysis of these country indicators can lead to different conclusions. Based on DDDs/1,000 patient days, antibiotic consumption in Belgian acute care hospitals is in line with countries those of the Netherlands, Denmark, and Sweden. However, based on DDDs/1,000 admissions, the use in Belgium is higher than in these countries. Country-specific hospital characteristics (differences in length of stay, antibiotic policies, duration and dosing of antibiotic treatment) might explain the differences in DDDs/1,000 admissions and DDDs/1,000 patient days. On the one hand, an advantage of expressing the consumption in DDDs/1,000 admissions is that this indicator is less influenced by the evolution towards shorter hospital stays than DDDs/1,000 patient days. On the other hand, DDDs/1,000 admissions are a less ideal indicator for analyses per hospital unit as misclassification bias can occur for patients who are admitted on different units during one hospital stay. Several investigators recommend the combination of different indicators [14,29]. Moreover, the hospital-specific indicators were successfully applied to analyse the antimicrobial selection pressure in hospitals and its relationship with specific outcomes [30,31]. DIDs cannot be used to express the consumption in individual hospitals and for the benchmarking between hospitals.

### Achievements and remaining challenges

Based on the current BeH-SAC surveillance, an improved reporting system (Healthstat.be) is used to provide both national and individualised reports of antimicrobial consumption per hospital. It also allows benchmarking with other comparable hospitals. On this interactive platform, users can choose their own parameters for the analyses: period (by year or by trimester), level of benchmarking (by kind (acute/chronic/psychiatric), type (primary, secondary or tertiary) or size of hospital), hospital units, antimicrobial agents (based on the ATC classification) and the denominator (by patient days, admissions, or no denominator to investigate the evolution of DDDs). Three types of reports are currently provided; in each, the consumption in the individual hospital can be analysed interactively together with the consumption rates in the benchmark group. All Belgian hospitals have access to this system.

The BeH-SAC surveillance could be further improved by not only reporting the consumption by hospital unit, but also by diagnosis (e.g. using ‘All Patient Refined Diagnostic Related Groups’ (APR-DRG) or ‘International Statistical Classification of Diseases and Related Health Problems’ (ICD)). This would help hospitals to better interpret the consumption rates according to their guidelines for antimicrobial treatment, and to define targets and action points to address inappropriate consumption [26]. Currently, the NIHDI provides reports of the antimicrobial consumption per APR-DRG to hospitals, but with a large delay in time (> 2 years) and without the possibility to benchmark [32]. A cooperation and integration of this system in BeH-SAC is being investigated.

Besides DDDs, a second indicator based on the recommended doses in Belgian acute care hospitals and adjusted to paediatric formulations (DDA: Daily Doses Administered) could also help hospitals with the interpretation of the results. As highlighted above, real-time feedback with an as small delay as possible should be integrated in the future. This is crucial for the targeted and timely management of an outbreak of multidrug-resistant bacteria [33]. Given that up to 2 years after the actual consumption corrections still can occur in the reimbursement data, finding the right balance between direct feedback with preliminary data and delayed feedback with more validated and corrected data remains challenging.

As far as we know, no countrywide actions have been initiated to address the variation in antibiotic consumption between hospitals and to investigate the reasons behind this variation. This can be considered as an action point for the future.

## Conclusion

In conclusion, antibiotic consumption in Belgian acute care hospitals remained overall stable over consecutive years despite several BAPCOC initiatives to lower the selection pressure for multidrug-resistant organisms. Especially the high variation in antibiotic consumption across Belgian acute care hospitals should further be investigated. Based on DDDs/1,000 patient days, the magnitude of antibiotic consumption is comparable with other European countries with a restrictive antibiotic policy (the Netherlands, Denmark, Sweden) while antibiotic consumption expressed in DDDs/1,000 admissions is higher. Our results underline the importance of examining the relationship of antibiotic consumption with patient-specific outcomes by using hospital-specific indicators to express antimicrobial consumption (besides DIDs) and combining different indicators.

Main advantages of the new methodology are the decreased registration load for the hospitals and the increased uniformity of the data collection across different hospitals. The data of the BeH-SAC surveillance can be used for benchmarking and for assessing interventions to improve antibiotic consumption in Belgian acute care hospitals. The methodology of the surveillance can further be improved with more detailed data per diagnosis and shorter delay (real-time feedback).

## Supplementary Data

702000 Supplementary Material
