# Supplementary material for: Antibiotic consumption in Belgian acute care hospitals: analysis of the surveillance methodology, consumption evolution 2003 to 2016 and future perspectives
Source: Euro Surveill. 2019 Nov 14;24(46):1900098. doi: 10.2807/1560-7917.ES.2019.24.46.1900098 (PMC6864973; doi:10.2807/1560-7917.ES.2019.24.46.1900098)

SUPPLEMENTARY MATERIAL

"This supplementary material is hosted by *Eurosurveillance* as supporting information alongside the article ‘Antibiotic consumption in Belgian acute care hospitals 2003-2016: analysis of the surveillance methodology, consumption evolution and future perspectives’ on behalf of the authors who remain responsible for the accuracy and appropriateness of the content. The same standards for ethics, copyright, attributions and permissions as for the article apply. Supplements are not edited by *Eurosurveillance* and the journal is not responsible for the maintenance of any links or email addresses provided therein."

Supplementary Table S1: Description of acute care hospitals in Belgium (2003-2016) included in BeH-SAC (Belgian Hospitals – Surveillance of Antimicrobial Consumption)

	2003	2004	2005	2006	2007	2008	2009	2010	2011	2012	2013	2014	2015	2016
**Total**	104	104	104	104	105	105	105	105	105	105	105	104	102	102
**Type:**														
Primary	80	80	80	80	80	80	80	80	80	80	80	79	77	77
Secondary	16	16	16	16	17	17	17	17	17	17	17	17	17	17
Tertiary	7	7	7	7	7	7	7	7	7	7	7	7	7	7
Specialized	1	1	1	1	1	1	1	1	1	1	1	1	1	1
**Size:**														
Large (>600 beds)	27	27	27	27	27	27	27	27	27	27	27	27	27	27
Medium (400-600 beds)	27	27	27	27	27	27	27	27	27	27	27	26	26	26
Small (<400 beds)	50	50	50	50	51	51	51	51	51	51	51	51	49	49
**Region:**														
Flanders	55	55	55	55	55	55	55	55	55	55	55	55	54	54
Wallonia	37	37	37	37	38	38	38	38	38	38	38	37	36	36
Brussels	12	12	12	12	12	12	12	12	12	12	12	12	12	12
**Mean length of stay**	NA*	NA*	NA*	NA*	NA*	7.72	7.44	7.38	7.24	7.12	6.95	6.92	6.84	6.66

* Number of admissions, needed to calculate the mean length of stay, only available for the period 2008-2016

Supplementary Table S2: A SWOT-analysis of the new surveillance methodology (BeH-SAC) based on reimbursement data

**Belgian Hospitals – Surveillance of Antimicrobial Consumption (BeH-SAC)**	
**Strengths**	**Weaknesses**
▪ Reuse of existing validated data collected for other purposes (reimbursement data)<br>▪ No registration load for hospitals<br>▪ Uniformity of the data collection<br>▪ Extended database (all hospitals, all patients with a hospital insurance (99% in Belgium))<br>▪ Detailed data on different levels (national, regional, hospital and unit)<br>▪ Use of different hospital-specific indicators (DDDs/1,000 patients days and DDDs/1,000 admissions) besides DIDs<br>▪ Interactive national and hospital-specific reports (Healthstat.be) with benchmarking between comparable hospitals<br>▪ Using the results for the antimicrobial selection pressure before a MDRO outbreak	▪ DDDs not always adjusted to the doses used in the local hospital setting<br>▪ DDDs not appropriate for paediatric use<br>▪ No DOT available<br>▪ Non-reimbursed antimicrobial consumption not included (but limited in Belgium)<br>▪ Large delay of the reimbursement data (> 1 year)<br>▪ Late adjustments in the data (until 2 years after first data delivery) can occur<br>▪ Currently no link with the indication<br>▪ Data not detailed enough to give feedback to specific prescribers (e.g. pneumologists, cardiologists)
**Opportunities**	**Threats**
▪ Adding a new indicator based on the recommended doses in Belgian acute care hospitals and adjusted to paediatric formulations (DDA)<br>▪ Linking antimicrobial consumption with diagnoses<br>▪ Comparison with other European countries based on hospital-specific indicators<br>▪ Inspiration for hospitals to set up a local surveillance system<br>▪ Using the results for the evaluation of antimicrobial stewardship programs<br>▪ Linking antimicrobial consumption data with antimicrobial resistance data on a hospital level<br>▪ Using the results for the evaluation of interventions during a MDRO outbreak	▪ Over/underestimations due to the limitations of DDDs<br>▪ Overestimation if not the whole drug unit was used (e.g. pharmaceutical compounding, individual dosing)<br>▪ Limited usability and relevance of old data<br>▪ Administrative modifications that do not accurately reflect clinical practices

DDA: daily dose administered; DDD: defined daily dose; DID: DDDs/1,000 inhabitants/day; DOT: days of treatment; MDRO: multidrug-resistant organisms.

Supplementary Figure S1: Evolution of the consumption of antibacterials for systemic use (J01) in intensive care units (ICU, blue) compared with the overall use in the hospital (Total, ICU included, red), expressed in defined daily doses (DDDs)/1000 patients days (acute care Belgian hospitals, 2003-2016)

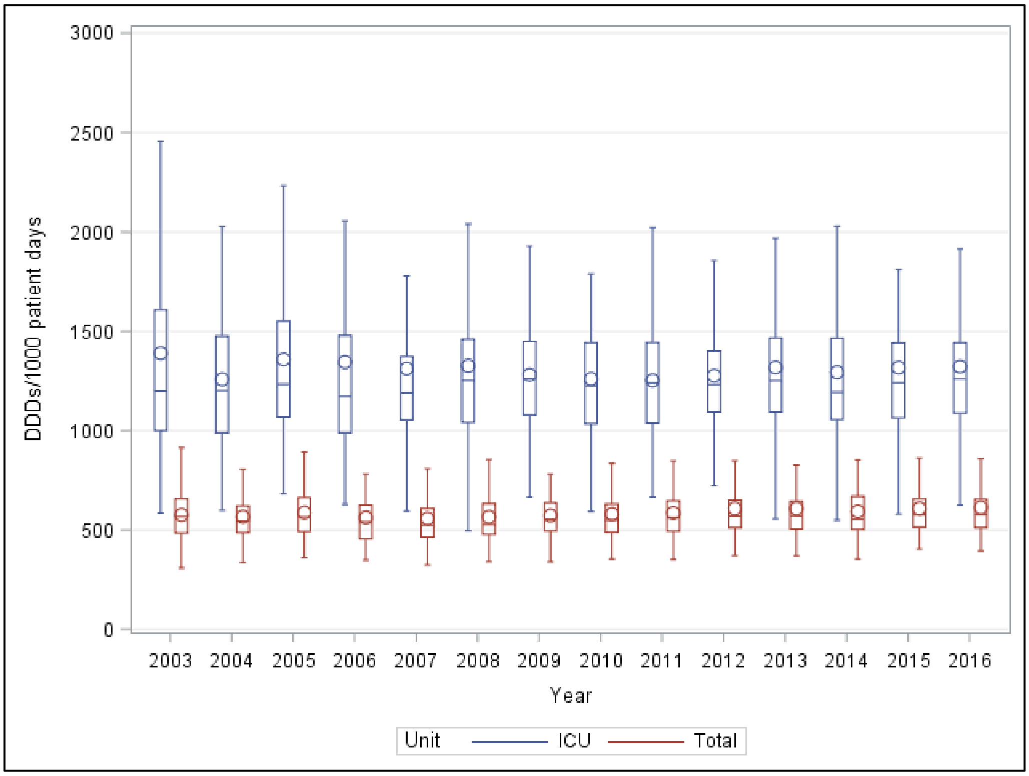

Legend boxplot: a. maximum (without outliers, 1.5x interquartile range), b. 75 percentile (P75), c. median, d. mean, e. 25 percentile (P25), f. minimum (without outliers, 1.5x interquartile range)

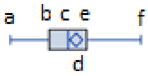

Supplementary Table S3: Median consumption of antibacterials for systemic use (J01) in 2016 per hospital unit (acute care Belgian hospitals), expressed in defined daily doses (DDDs)/1000 patient days

	Median consumption in DDDs/1000 patient days in 2016	Number of hospitals included
**Intensive care**	1261.0	101
**Burn unit**	740.4	5
**Pediatrics**	682.2*	94
**Internal medicine (including infectious diseases)**	658.0	102
**Surgery**	646.2	102
**Geriatrics**	510.0	98
**Specialized care: cardio-pulmonary**	277.7	16
**Maternity**	242.7	96
**Specialized care: chronic - polypathology**	206.0	19
**Specialized care: locomotive**	177.1	65
**Specialized care: neurological**	154.0	24
**Specialized care: chronic - palliative care**	125.7	45
**Neonatology, intensive care**	117.5*	19
**Specialized care: psycho-geriatrics**	104.4	15
**Neonatology, non-intensive**	50.3*	80
**Overall consumption in the hospital**	577.1	102

* DDDs have been developed for adults (70 kg) so interpretation for new-borns, infants and children is therefore not straightforward.

Supplementary Figure S2: Evolution of the consumption of antibacterials for systemic use (J01) per type of hospital (primary=blue, secondary=red, tertiary=green), expressed in defined daily doses (DDDs)/1000 patient days (acute care Belgian hospitals, 2010-2016)

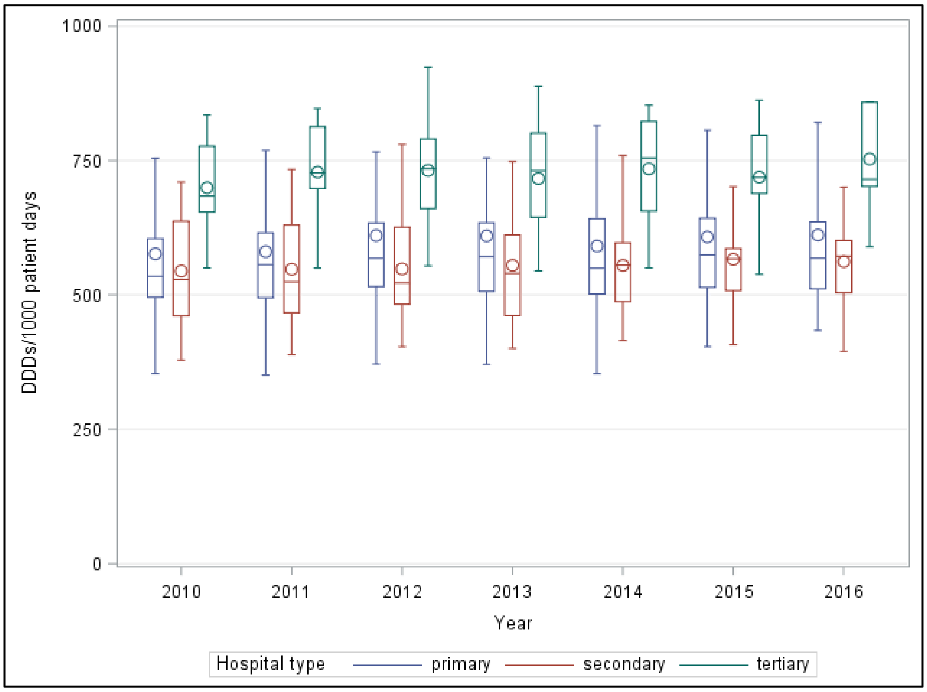

Legend boxplot: a. maximum (without outliers, 1.5x interquartile range), b. 75 percentile (P75), c. median, d. mean, e. 25 percentile (P25), f. minimum (without outliers, 1.5x interquartile range)

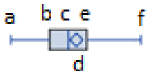

Supplementary Figure S3: Evolution of the consumption of antibacterials for systemic use (J01) per region (Brussels = blue, Flanders = red, Wallonia = green), expressed in defined daily doses (DDDs)/1000 patient days (acute care Belgian hospitals, 2010-2016)

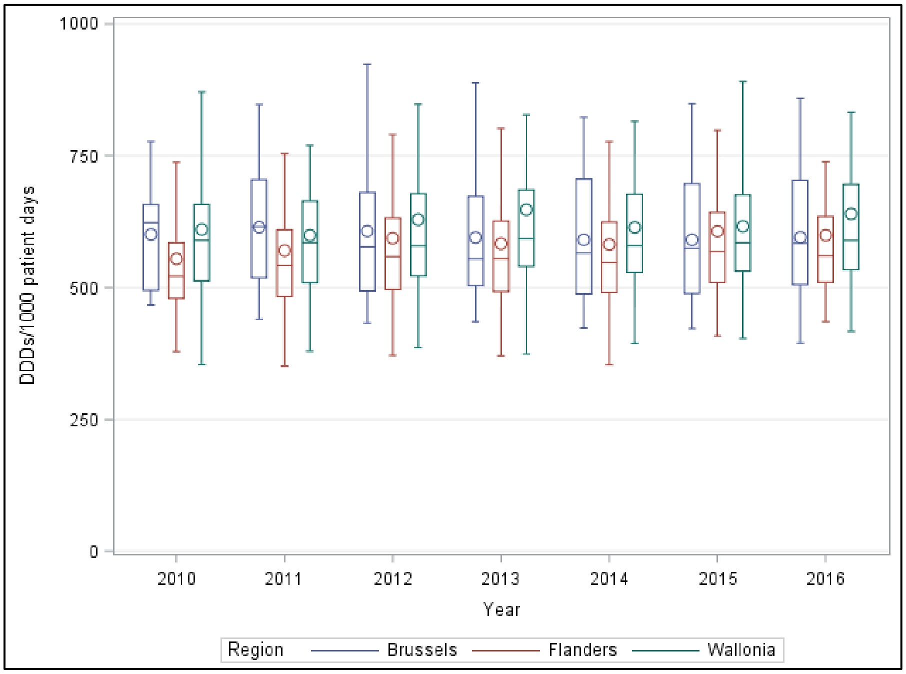

Legend boxplot: a. maximum (without outliers, 1.5x interquartile range), b. 75 percentile (P75), c. median, d. mean, e. 25 percentile (P25), f. minimum (without outliers, 1.5x interquartile range)

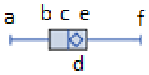

Supplementary Figure S4: Evolution of the consumption of antibacterials for systemic use (J01) per hospital size (<400 beds: small = green, 400-600 beds: medium = red, >600 beds: large = blue), expressed in defined daily doses (DDDs)/1000 patients days (acute care Belgian hospitals, 2010-2016)

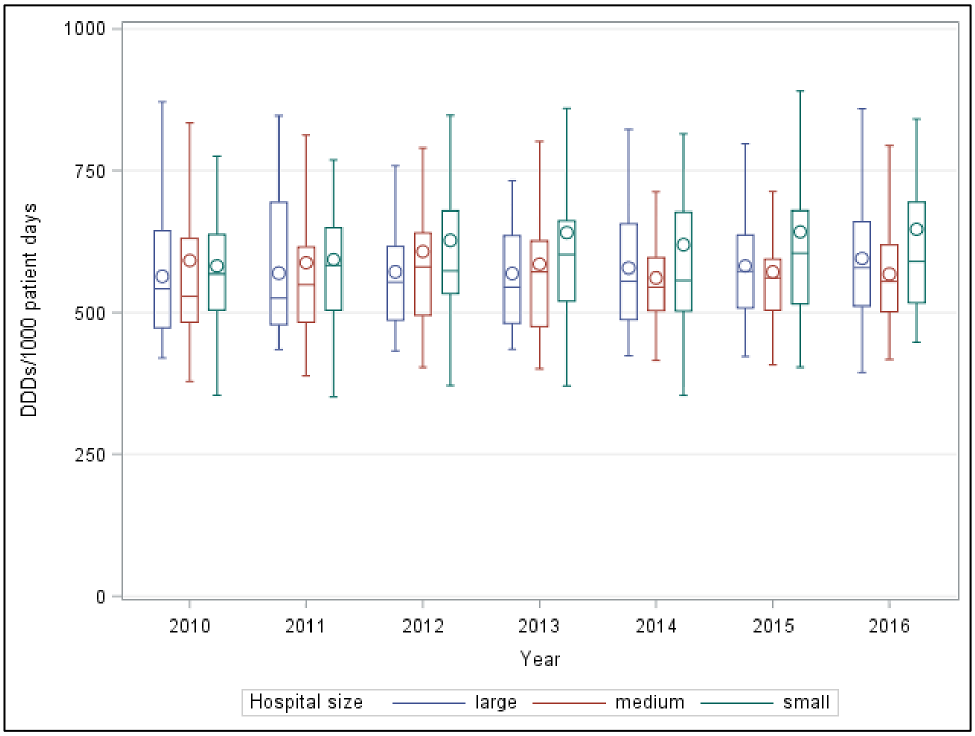

Legend boxplot: a. maximum (without outliers, 1.5x interquartile range), b. 75 percentile (P75), c. median, d. mean, e. 25 percentile (P25), f. minimum (without outliers, 1.5x interquartile range)

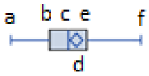

Supplementary Figure S5: Stacked bar plot with the evolution (2003-2016) of the median consumption of the most important antibiotic subclasses (expressed in defined daily doses (DDDs)/1000 patient days) in acute care Belgian hospitals

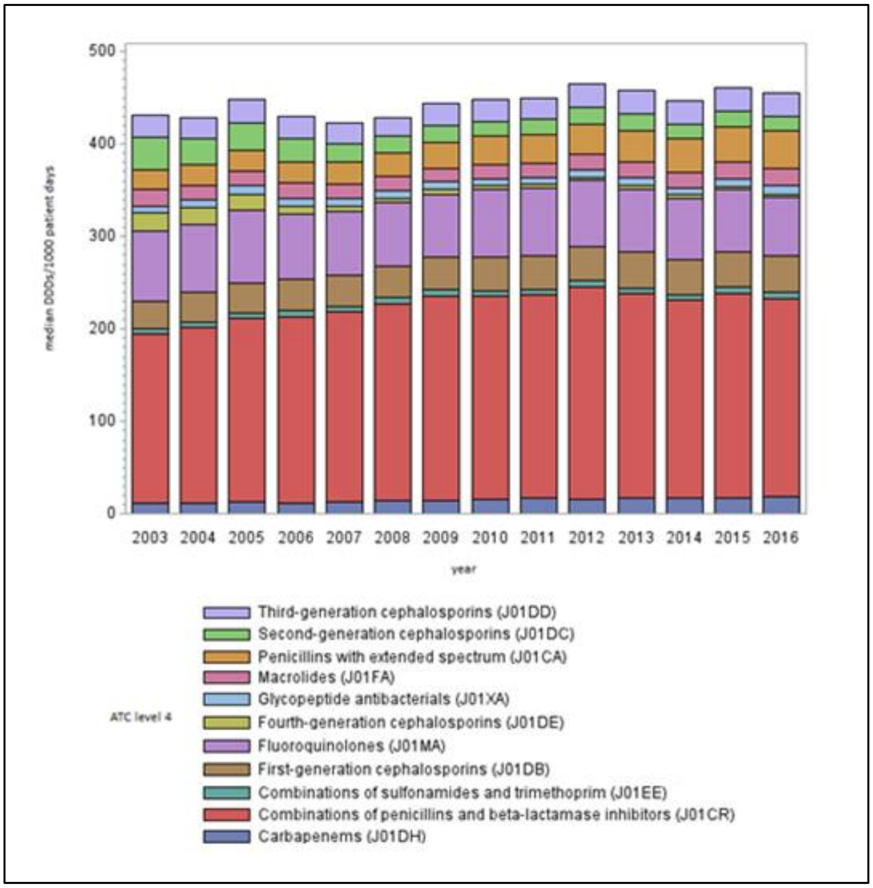

Supplement: Supplementary Material [file 19-00098_VANDAEL_SupplementaryMaterial.pdf]
